# Sodium-Glucose Cotransporter-2 Inhibitors and Euglycemic Diabetic Ketoacidosis: A Case Series of Three Post-surgical Patients

**DOI:** 10.7759/cureus.84665

**Published:** 2025-05-23

**Authors:** Daniela Z Bazan, Lauren Esqueda, Ashly Ibrahim, Lorena Gonzalez, Jeffrey J Skubic, John Reilly, Ramiro Cavazos, Rene Verduzco

**Affiliations:** 1 Pharmacy Practice, Texas A&M University, Edinburg, USA; 2 Pharmacy, Doctors Hospital at Renaissance, Edinburg, USA; 3 Pharmacy, Texas A&M University, Kingsville, USA; 4 Surgical Critical Care and Trauma Surgery, Doctors Hospital at Renaissance, Edinburg, USA; 5 Surgery, Doctors Hospital at Renaissance, Edinburg, USA

**Keywords:** case series, clinical pharmacology, euglycemic diabetic ketoacidosis, peri-operative management, sodium-glucose cotransporter 2 inhibitor, type 2 diabetes mellites

## Abstract

Sodium-glucose cotransporter 2 (SGLT2) inhibitors are a class of commonly prescribed medications used in diabetes management for both their cardiovascular and renal benefits. However, as a class, SGLT2 inhibitors have a known side effect risk for ketoacidosis, including the rarer version called euglycemic diabetic ketoacidosis (DKA). We present three cases of euglycemic DKA postoperatively, in patients who were on SGLT2 inhibitors for type 2 diabetes mellitus. A DKA protocol was initiated for all three patients, and after treatment with fluid and insulin therapy, all patients recovered. All three were restarted on their SGLT2 inhibitor upon discharge. Based on this limited experience, we recommend close monitoring of postoperative patients on SGLT2 inhibitors to allow early detection of euglycemic DKA. Larger trials are needed to determine the exact, optimal timing of restarting SGLT2 inhibitors postoperatively.

## Introduction

Sodium-glucose cotransporter 2 (SGLT2) inhibitors are a class of medications used to treat type 2 diabetes mellitus (T2DM) by blocking the reabsorption of glucose in the kidneys, resulting in increased urinary glucose excretion and improved glycemic control [1]. There are currently five SGLT2 inhibitors approved by the Federal Food and Drug Administration (FDA): canagliflozin (Invokana^®^), dapagliflozin (Farxiga^®^), empagliflozin (Jardiance^®^), ertugliflozin (Steglatro^®^), and bexagliflozin (Brenzavvy^®^). SGLT2 inhibitors have gained popularity in recent years due to their cardiovascular and renal benefits [2-5]. SGLT2 inhibitors have been shown to reduce cardiovascular events, such as myocardial infarction, stroke, and cardiovascular death [2]. They can lower heart failure hospitalizations and reduce the progression of kidney disease [6]. These benefits have been shown in patients with or without T2DM, so the FDA has expanded the indications to include chronic kidney disease and heart failure. SGLT2 inhibitors can cause genital mycotic infections, polyuria, dehydration, dizziness, or hypotension. Post-marketing analysis raised safety concerns about the increased risk of euglycemic diabetic ketoacidosis (DKA) [7]. The risk is higher in patients with increased insulin needs, such as those with acute illness, infection, surgery, or trauma. Risk is also increased in patients with low carbohydrate intake or dehydration, which makes post-surgical patients more vulnerable to euglycemic DKA [8]. In 2020, the FDA updated the labeling for SGLT2 inhibitors and added a warning and precaution for increased risk of euglycemic DKA. In 2022, to lessen the risk, it was modified to state that healthcare professionals should consider stopping canagliflozin, dapagliflozin, bexagliflozin, and empagliflozin at least three days before a scheduled surgery, and stop ertugliflozin four days before surgery [7,9].

Euglycemic DKA and SGLT2 inhibitors

DKA occurs when the body increases the formation of new glucose molecules, also known as gluconeogenesis, and decreases the utilization of glucose by the peripheral tissues. There is also an increase in the production of the counter-regulatory hormone glucagon, which stimulates hepatic gluconeogenesis and the production of ketones, or ketogenesis. Hyperglycemia, combined with insulin deficiency, leads to increased hepatic ketone production and lipolysis from adipose tissue. Lipolysis is the metabolic process where triglycerides are broken down into glycerol and free fatty acids. The excess free fatty acids are transported to the liver and converted to ketone bodies. Ketone bodies are acidic and contribute to the metabolic acidosis seen in DKA [10,11]. Euglycemic DKA is a rare but serious adverse effect of SGLT2 inhibitors, characterized by the presence of DKA with plasma glucose that is normal or only slightly elevated [7,12]. Euglycemic DKA can be difficult to diagnose and can cause delays in treatment because blood glucose levels may be normal or only mildly elevated (<250 mg/dL) [13]. Signs and symptoms at presentation are consistent with severe metabolic acidosis and include nausea, vomiting, abdominal pain, generalized malaise, and shortness of breath [7,13]. The association between SGLT2 inhibitors and euglycemic DKA is not fully understood, but it is thought to be related to the reduction of insulin combined with glucagon release, poor glucose availability, ketone body production, and urinary glucose excretion [13]. By inducing glycosuria, SGLT2 inhibitors cause osmotic diuresis and dehydration. This triggers the synthesis of glucagon, cortisol, and adrenaline, which contribute to lipolysis and ketogenesis. There are additional factors that put patients at an increased risk for developing euglycemic DKA. These include a reduction of insulin dose, acute febrile illness, reduced caloric intake due to illness or surgery, pancreatic disorders resulting in insulin deficiency, and alcohol abuse [7].

## Case presentation

Here we present a case series of three different patients (Table 1) taking an SGLT2 inhibitor for T2DM, who were admitted to the hospital for surgeries and developed euglycemic DKA during their stay. At this institution, DKA is diagnosed as a pH less than 7.3, plasma glucose greater than 250 mg/dL, an anion gap greater than 10 mEq/L, serum bicarbonate less than 18 mEq/L, and/or the presence of serum/urine ketones. A patient’s DKA is considered resolved when the pH is greater than 7.3, blood glucose is less than 200 mg/dL, the anion gap is less than or equal to 12 mEq/L, and/or the serum HCO₃ level is greater than 18 mEq/L (Table 2).

**Table 1:** Patient Characteristics Abbreviations: HbA1c, hemoglobin A1c; T2DM, type 2 diabetes mellitus; CAD, coronary artery disease; GERD, gastroesophageal reflux disease; SGLT2, sodium-glucose cotransporter-2; DKA, diabetic ketoacidosis; ICU, intensive care unit

	Case 1	Case 2	Case 3
Age/sex	68/female	66/male	55/female
Admission weight (kg)	77	105	62
HbA1c (%)	7.3	7.7	7.7
Admission diagnosis	Adenocarcinoma of gallbladder	Left hip fracture	Sepsis and pelvic mass
Comorbidities	T2DM, Hypertension, Hyperlipidemia, CAD, GERD	Hypertension, T2DM	T2DM, Hypertension, Hyperlipidemia, Obesity, Carcinosarcoma of uterus, Peripheral vascular disease
Home medications for T2DM	Metformin	Metformin, Semaglutide, Gliclazide	Metformin, Semaglutide
SGLT2 inhibitor	Empagliflozin	Canagliflozin	Empagliflozin
Time SGLT2 inhibitor held before surgery (days)	1	Unknown	Unknown
Time from surgery to DKA diagnosis (hours)	51	72	4
Length of stay in ICU (days)	7	7	6
Total length of stay (days)	10	18	9
Time to resolution of EDKA (days)	6	2	2
Discharge T2DM medications	Empagliflozin, Metformin	Canagliflozin, Metformin, Semaglutide, Gliclazide	Empagliflozin, Metformin, Semaglutide

**Table 2:** Laboratory Values Abbreviations: DKA, diabetic ketoacidosis; POD, postoperative day

Lab	Normal ranges	DKA diagnosis			DKA (day after diagnosis)			DKA resolution		
Case and POD		Case 1 POD 2	Case 2 POD 3	Case 3 POD 0	Case 1 POD 3	Case 2 POD 4	Case 3 POD 1	Case 1 POD 4	Case 2 POD 5	Case 3 POD 2
Glucose (mg/dL)	74-109	118	163	275	152	164	240	158	164	171
HCO_3_^-^ (mmol/L)	22-29	14	23	6	6	17	20	17	20	20
Anion gap (mEq/L)	NA	26	12	22	13	13	19	7	9	8
pH	7.35-7.45	7.39	7.4	6.86	7.41	7.47	7.4	7.43	7.47	7.44
PCO_2_ (mmHg)	35-45	20	33	37	29	30	24	31	30	34

Case 1

A 68-year-old Hispanic woman, weighing 77 kg and with a height of 153 cm, presented to the hospital for a liver resection of segments IVb and V, following a recent diagnosis of gallbladder adenocarcinoma. She had recently undergone a laparoscopic cholecystectomy three weeks prior to admission. The patient had a past medical history significant for T2DM, gastroesophageal reflux disease (GERD), hypertension, hyperlipidemia, coronary artery disease (CAD), and adenocarcinoma of the gallbladder. The patient's active home medications for the treatment of T2DM were empagliflozin 25 mg orally once daily and metformin 850 mg orally once daily. There were no reported medication allergies, and the patient's renal function was normal. This case was scheduled, and the patient stated she had last taken empagliflozin the day before surgery.

The patient was admitted to the intensive care unit (ICU) after surgery for recovery. On the morning of postoperative day (POD) 1, labs were drawn, and she was noted to be hyperglycemic (232 mg/dL), with an anion gap of 16 mEq/L, serum bicarbonate of 19 mmol/L, and pH 7.32. On POD 2, approximately 51 hours after the surgery, she began to experience abdominal pain and increased shortness of breath, with an SpO₂ as low as 89 and requiring 3-4 liters (L) of oxygen. She was started on bilevel positive airway pressure (BiPAP), and a heparin infusion was initiated while a computed tomography (CT) scan of the chest was ordered to rule out pulmonary embolism. At the time of diagnosis, her serum glucose was 118 mg/dL, with an elevated anion gap of 29 mEq/L, serum bicarbonate of 14 mmol/L, and pH of 7.39. Ketones were present in the urine, and a serum acetone level was drawn and found to be positive. The team started an intravenous (IV) insulin infusion and IV fluids per the institution’s DKA protocol. The anion gap closed (12 mEq/L) on POD 3, and the insulin infusion was stopped. That evening, the anion gap re-opened (18 mEq/L), and the IV insulin infusion was restarted. The insulin infusion continued until POD 7, when the gap closed (13 mEq/L), and the patient was downgraded to a medical floor. The patient was discharged home on POD 9 on the same medications for the treatment of her T2DM, including empagliflozin 25 mg orally once daily.

Case 2

A 66-year-old White male, weighing 105 kg and with a height of 193 cm, presented to the hospital as a transfer from another facility for further treatment of a left hip fracture. The patient is a truck driver who fell two feet out of his semi-truck three days prior to admission. The patient’s chief complaint was left hip and leg pain, along with an inability to walk. His past medical history included hypertension and T2DM. The patient’s diabetes was chronically managed with canagliflozin 100 mg orally once daily, metformin 1000 mg orally twice daily, semaglutide 1 mg subcutaneously once weekly, and gliclazide 60 mg orally once daily. The patient had no known drug allergies, and renal function was normal. The patient was ordered to be kept on nothing by mouth (NPO) status and underwent a left hip hemiarthroplasty the day after admission. The patient underwent surgery with no complications. Due to the emergent nature of the surgery, the timing of the patient’s last canagliflozin dose could not be confirmed through medication history or records.

On POD 1, the patient was tolerating diet and had no complaints, despite an anion gap of 14 mEq/L and serum bicarbonate of 21 mmol/L. Overnight on POD 1, the patient experienced dyspnea and was put on 15 L of oxygen. On POD 2, the patient was weaned to 3 L of oxygen. A CT scan of the chest was completed to rule out pulmonary embolism. On POD 3, the patient was transferred to the surgical intensive care unit (SICU) after further dyspnea and episodes of vomiting. The patient was tachycardic, hypertensive, febrile, and experiencing abdominal pain. The patient was no longer able to tolerate oral intake. The diagnosis of euglycemic DKA was made on POD 3, approximately 72 hours after surgery, with a glucose of 163 mg/dL, serum bicarbonate of 23 mmol/L, an anion gap of 12 mEq/L, respiratory compensation (PCO₂ 33 mmHg), and ketones present in the urine. The patient was initiated on the facility’s DKA protocol and started on an IV insulin infusion and fluids. Over the next two days, symptoms improved as the anion gap closed; diet was advanced as nausea and vomiting improved. The patient’s anion gap closed on POD 5, with a gap of 9 mEq/L, and DKA was diagnosed as resolved, with a glucose of 144 mg/dL, serum bicarbonate of 23 mmol/L, and a closed anion gap. The patient remained on an IV insulin infusion to clear glucose from the urine until POD 8 and was then transitioned to subcutaneous insulin. On POD 9, the patient was downgraded to a medical floor and remained off the IV insulin infusion, with the anion gap still closed. The patient was discharged to an inpatient rehabilitation center on POD 16 on the same diabetes medications, including canagliflozin 100 mg orally once daily.

Case 3

A 55-year-old Hispanic female, weighing 62 kg and with a height of 155 cm, presented to our facility for severe right lower quadrant pain. The patient was experiencing nausea and vomiting, eye pain, and fever. The patient’s initial CT scan of the abdomen showed a cystic structure in the right lower quadrant with inflammatory changes. The patient was admitted for sepsis and a pelvic mass. The patient had a past medical history of hypertension, hyperlipidemia, obesity, carcinosarcoma of the uterus, peripheral vascular disease, and T2DM. The T2DM was managed with empagliflozin 25 mg orally once daily, metformin 1000 mg orally twice daily, and semaglutide 0.25 mg subcutaneously once weekly. The patient had no known drug allergies, and renal function was normal. This was not an elective or scheduled surgery, so the status of the last dose taken of empagliflozin is unknown.

The patient was sent to the operating room (OR) for a diagnostic laparoscopy after increasing abdominal pain. During surgery, the patient underwent an appendectomy, cystectomy, and ligation of a muscle bleed. The patient developed discoloration of the right leg after ligation of the bleed. The decision was made to perform an angiogram of the pelvis and right leg while the patient was still in the OR. The patient underwent vascular repair and revascularization of the arteries. There were no complications during the surgery. Approximately four hours after surgery on POD 0, the patient was diagnosed with euglycemic DKA. The patient experienced metabolic acidosis, with an anion gap of 22 mEq/L, serum bicarbonate of 6 mmol/L, pH of 6.86, and respiratory compensation with a PCO₂ of 37 mmHg. Ketones were present in the urine, and serum acetone was positive. The patient was transferred to the SICU, the DKA protocol was initiated, and the patient was started on an IV insulin drip and IV fluids. The patient was placed on a mechanical ventilator and was tachycardic. On POD 1, the insulin drip was paused due to hypophosphatemia. Electrolytes were replaced per the DKA protocol, and the patient was restarted on the insulin infusion. The patient’s anion gap remained elevated at 19 mEq/L until later that night, when it closed at 10 mEq/L. On POD 2, the patient’s anion gap remained closed at 8 mEq/L. The patient’s DKA was resolved, with a glucose of 171 mg/dL, serum bicarbonate of 24 mmol/L, pH of 7.44, and a closed anion gap. The patient was taken off the continuous insulin infusion and started on intermittent subcutaneous insulin. On POD 3, the patient was extubated, and diet was advanced. The patient was discharged on POD 6 on the same diabetes regimen, including empagliflozin 25 mg orally once daily and semaglutide, with instructions to increase to 0.5 mg subcutaneously after four weeks.

## Discussion

A literature search was conducted to find case reports of euglycemic DKA associated with SGLT2 inhibitor use, specifically during the perioperative stage (Table 3). We presented three patient cases of euglycemic DKA after surgery at our institution. The diagnosis of euglycemic DKA varied in each case, from zero to three days postoperatively, which was consistent with other cases of euglycemic DKA after surgery, with many cases beginning hours to a few days after surgery.

**Table 3:** Literature Search: Case Reports Abbreviations: SGLT2, sodium-glucose cotransporter-2; DKA, diabetic ketoacidosis; POD, post operative day; NPO, nothing by mouth

Study	SGLT2 inhibitor	Age and sex	Surgery	Onset of DKA (time)	Resolution of DKA (time)	Clinical presentation	Predisposing factors of DKA	Medication stopped prior to surgery (time)
Wang and Isom (2020) [8]	Empagliflozin	40, female	Cerebral revascularization for moyamoya disease	24 hours	Unknown	Elevated anion gap, metabolic acidosis	Surgical stress, acute post-operative illness, decreased carbohydrate intake	Yes (at least 18 hours)
Osafehinti et al. (2021) [14]	Empagliflozin	60, male	Coronary artery bypass graft	Few hours	2 days	Elevated anion gap, metabolic acidosis	Surgery	Yes (48 hours)
Gomez-Sanchez et al. (2021) [15]	Empagliflozin	73, male	Left femoral endarterectomy	POD 2	POD 3/4	Acute delirium, severe metabolic acidosis, anion gap, hypotensive	None noted	Morning of surgery (exact time unknown)
Ritchie and Dixon (2022) [16]	Empagliflozin	Early 60s, male	Total hip arthroplasty	Morning of surgery (unclear) (3 days of on and off fasting)	4 days	Severe anion gap, metabolic acidosis	Decreased carbohydrate intake	No
Smith et al. (2021) [17]	Canagliflozin	51, female	Laparoscopic sleeve gastrectomy	72 hours	4 days	Tachycardic, lethargic, tachypnea, metabolic acidosis, anion gap	NPO before surgery, bariatric surgery	Yes (48 hours)
Dizon et al. (2017) [18]	Canagliflozin	54, male	Laparoscopic cholecystectomy	POD 3	Unknown	Shortness of breath	Restarted on oral antidiabetics POD 1	Unknown

To assess whether the causality of the euglycemic DKA was due to SGLT2 inhibitor use, we utilized the Adverse Drug Reaction Probability Scale (Naranjo Scale). Both Case 1 and Case 2 received a score of 7, meaning the SGLT2 inhibitor was the probable cause of their euglycemic DKA. Case 3 received a score of 4, meaning the SGLT2 inhibitor was a possible cause of the patient’s euglycemic DKA. There were no other factors that might have caused the DKA in Cases 1 or 2, such as sepsis or nonadherence to insulin. Case 3 received a lower score due to the patient having sepsis present on admission, as that may have also contributed to their euglycemic DKA [19].

The 2024 American Diabetes Association (ADA) guidelines have recommendations for perioperative care in diabetes patients that include having a goal HbA1c of less than 8%, and to hold SGLT2 inhibitors at least three days before scheduled surgeries for canagliflozin, dapagliflozin, bexagliflozin, and empagliflozin, and four days for ertugliflozin [20]. In these three cases, the HbA1c of the patients was less than 8%. However, Case 1 was the only elective surgery in this case series, and the SGLT2 inhibitor was only held for one day prior to surgery, compared to the recommended three days. Dutta et al. (2022) conducted a systematic review and meta-analysis of all available case reports and clinical trial information and found that surgery was the highest risk factor for developing euglycemic DKA for patients on an SGLT2 inhibitor, and that this adverse effect is most common with canagliflozin [21]. Murugesan et al. (2022) looked specifically at patients undergoing cardiac surgeries, and of the 24 patients evaluated, 17 developed euglycemic DKA secondary to SGLT2 inhibitor exposure [22]. Euglycemic DKA related to SGLT2 inhibitors is a rare adverse effect, but it has now been established that surgery is a significant contributing factor. A learning point from these experiences is the need to closely monitor patients when the last exposure to an SGLT2 inhibitor is unknown. This occurs commonly in emergent surgeries, and while not much may be done at the time of the procedure, close monitoring right after surgery may lead to early detection and treatment of euglycemic DKA, and therefore decrease the likelihood of subsequent complications. Case 3 was the only case to have lab values taken on the morning of surgery. The blood glucose levels of Cases 2 and 3 were unable to be determined, as they were not drawn within the four hours prior to surgery as recommended. Case 2 also did not have labs drawn until POD 1, which may have delayed establishing the diagnosis. Additionally, all three cases had elevated anion gaps before official DKA diagnosis. Lab values of patients on SGLT2 inhibitors after surgery should be monitored closely for signs of metabolic acidosis. Euglycemic DKA is more difficult to diagnose since glucose is only mildly elevated, and can lead to a delay in treatment. All three patients were kept six to seven days in the ICU until the euglycemic DKA could be resolved, notably increasing each patient’s length of stay.

There are currently no recommendations for restarting the SGLT2 inhibitor after the incidence of euglycemic DKA. In our cases, the patients were restarted on their SGLT2 inhibitors at discharge, along with their other diabetic home medications, without any known issues. Some of the case reports reviewed did not specify whether the SGLT2 inhibitor was continued after discharge. From the reviewed cases that stated the outcome, all except one discontinued the SGLT2 inhibitor in their patients. The case that continued with the SGLT2 inhibitor gave the patient instructions to discontinue the medication three days before procedures. In the cases where the SGLT2 inhibitor was discontinued, most patients were started on basal and bolus insulin regimens [14-18]. In the case series by Chaudhry et al. (2022), the institution holds the SGLT2 inhibitor for at least one month after surgical discharge to allow the patient to fully recover [23]. Patients can restart the SGLT2 inhibitor if they are deemed clinically stable, with no risk factors for DKA, by their endocrinologist or other provider who manages their diabetes medications. This may be a viable recommendation, as the ADA guidelines suggest follow-up visits for diabetic patients within one month of discharge from an inpatient visit [20].

A limitation of this case series was that the patients’ records were accessed retrospectively. The information relied on the progress notes and laboratory data while the patients were admitted to our institution, so the accuracy of the information is limited to the accuracy of the recordkeeping by those following the patients at that time. Another limitation is the small sample size within one institution.

## Conclusions

The three cases presented portrayed incidences of euglycemic DKA occurring after three different types of surgeries. The increased risk for euglycemic DKA after surgery supports the recommendation to increase monitoring of patients on an SGLT2 inhibitor preoperatively, whether elective or not, to assist in the earlier diagnosis of patients. There is a paucity of formal recommendations for when to restart SGLT2 inhibitors after an episode of euglycemic DKA. Although our case series suggests that SGLT2 inhibitors can be safely restarted upon discharge after successful treatment of euglycemic DKA, larger trials are needed to confirm this and to determine the optimal timing for restarting SGLT2 inhibitors.
